# The requirements and challenges in preventing of road traffic injury in Iran. A qualitative study

**DOI:** 10.1186/1471-2458-9-486

**Published:** 2009-12-23

**Authors:** Davoud Khorasani-Zavreh, Reza Mohammadi, Hamid Reza Khankeh, Lucie Laflamme, Ali Bikmoradi, Bo JA Haglund

**Affiliations:** 1 Division of Social Medicine, Department of Public Health Sciences, Karolinska Institutet, Norrbacka, SE-171 76, Stockholm, Sweden; 2 Division of International Health Care Research (IHCAR), Department of Public Health Sciences, Karolinska Institutet, SE-171 77, Stockholm, Sweden; 3 National Public Health Management Centre (NPMC), Tabriz University of Medical Sciences, Tabriz, Islamic Republic of Iran; 4 Urmia University of Medical Sciences, Oroumiyeh, Islamic Republic of Iran; 5 Sina Trauma Research Center, Tehran University of Medical Sciences, Tehran, Islamic Republic of Iran; 6 Department of Nursing, University of Social Welfare and Rehabilitation, Tehran, Islamic Republic of Iran; 7 Medical Management Centre, Department of LIME, Karolinska Institutet, SE 171 77, Stockholm, Sweden; 8 Hamadan University of Medical Sciences, Hamadan, Islamic Republic of Iran

## Abstract

**Background:**

Road traffic injuries (RTIs) are a major public health problem, especially in low- and middle-income countries. Among middle-income countries, Iran has one of the highest mortality rates from RTIs. Action is critical to combat this major public health problem. Stakeholders involved in RTI control are of key importance and their perceptions of barriers and facilitators are a vital source of knowledge. The aim of this study was to explore barriers to the prevention of RTIs and provide appropriate suggestions for prevention, based on the perceptions of stakeholders, victims and road-users as regards RTIs.

**Methods:**

Thirty-eight semi-structured interviews were conducted with informants in the field of RTI prevention including: police officers; public health professionals; experts from the road administrators; representatives from the General Governor, the car industry, firefighters; experts from Emergency Medical Service and the Red Crescent; and some motorcyclists and car drivers as well as victims of RTIs. A qualitative approach using grounded theory method was employed to analyze the material gathered.

**Results:**

The core variable was identified as "The lack of a system approach to road-user safety". The following barriers in relation to RTI prevention were identified as: human factors; transportation system; and organizational coordination. Suggestions for improvement included education (for the general public and targeted group training), more effective legislation, more rigorous law enforcement, improved engineering in road infrastructure, and an integrated organization to supervise and coordinate preventive activities.

**Conclusion:**

The major barriers identified in this study were human factors and efforts to change human behaviour were suggested by means of public education campaigns and stricter law enforcement. However, the lack of a system approach to RTI prevention was also an important concern. There is an urgent need for both an integrated system to coordinate RTI activities and prevention and a major change in stakeholders' attitudes towards RTI prevention. The focus of all activities should take place on road users' safety.

## Background

Road traffic injuries (RTIs) are a major global public health problem, requiring concerted efforts [1]. Without increased efforts and activities, it is expected that the total number of injuries will rise by 65% between 2000 and 2020 and deaths are expected to increase by more than 80% [2-4]. This burden is mainly related to low- and middle-income countries (LMICs) [1,5-7]. The number of injuries and deaths also depends largely on changes to the transportation infrastructure [8].

In order to make a road traffic system safer, understanding the system as a whole and the interaction between its elements (including vehicles, roads and road-users along with their physical, social and environmental circumstances) is important. It is also crucial to identify where there is potential for intervention [9]. In LMICs, where the burden of RTIs is the heaviest, there is little or no public health leadership for the prevention and control of their consequences [10]. Provision of safely designed roads and vehicles is necessary to achieve low road fatality rates, but this alone is not sufficient [7]. However, because of a large variety of contextual differences, like road traffic environments and cultural aspects, the risks encountered may vary considerably among different countries [11].

Data from the World Health Organization (WHO) reveal that Iran has one of the highest mortality rates from RTIs in the world [12]. In order to deal with this, the Iranian parliament decided on a national policy to reduce fatal RTIs in 2004. A large number of traffic-related injury policy interventions and strategies in high-income countries can be potentially transferable to LMICs. However, it is important to consider that country-specific factors such as national culture, feasibility, and barriers for policy implementation must be considered [13]. A review of the literature revealed that most published studies from Iran examined RTIs using mainly an epidemiological approach [12,14-17]. The interaction between the components and a better understanding of the phenomena are crucial. The few in-depth qualitative studies available [18-22] have focused on specific groups of road-users and were limited in scope. These studies do not provide much information as to various stakeholders' perceptions regarding what are the barriers in RTIs prevention and how the current situation can be improved. However, stakeholders' perceptions are indeed important for quality improvement [23-26].

Thus, in order to find the essence of the phenomena, it was found fruitful, as one important step, to explore the phenomena in more depth. The present article therefore was designed to explore informants' perceptions on the factors obstructing RTI prevention and to identify facilitators that could lead to greater road-user safety in Iran.

## Methods

The study was performed using grounded theory method. Grounded theory is a suitable method when researchers wish to explore a known area from a fresh perspective [27-29]. This article is part of a larger study that has explored pre-crash and crash situation.

### Study setting

This study was conducted in 2007 among informants in West Azarbaijan Province and at the national level in Iran, interviewing those involved in RTI control, other road-users and victims of RTIs. In Iran there are several organizations involved in RTI prevention (See below).

### Study participants and data collection

The principal investigator, who has work experience in the country in post-crash issues (e.g., victim management), took responsibility to contact eligible key persons in different organizations. Accordingly, stakeholders who have experience and knowledge, representing their perspectives on RTIs, were approached for interviews. A few victims and road-users were also interviewed because of the unique perspective they could add on what takes place in the field. The thirty-eight participants included: six police officers; five public health professionals; two experts from the Ministry of Road; and four experts from the Road & Transportation Office; three representatives of the General Governor of West Azarbaijan Province, the car industry and firefighters; seven staff from the Emergency Medical Services; and two from Red Crescent; four road-users including two motorcyclists and two car drivers; and five victims of RTIs. The number of participants was determined based on saturation principles. Their ages ranged between 20 and 65, and their education ranged from nine-year intermediate school to professional education in the field of medicine.

Semi-structured interviews in Persian were used for data collection. We were looking to gather informants' perspectives from different groups and organizations and this procedure felt far more suitable. They began with simple and general questions, gradually progressing to more specific ones. Probing was performed according to the reflections of each participant, concerning experiences of RTIs, the barriers to RTI prevention, opinions about barriers related to transport system, perceptions about road-users' behaviour as well as planning and organizational coordination. Interviews lasted between 45 and 80 minutes. Theoretical sampling was also used to contact other key informants; those who weren't considered initially as participants in order to saturate data and all generated concepts. By theoretical sampling, we refer to a sampling procedure of high value in qualitative research, especially in the grounded theory [28]. The goal of theoretical sampling is to gain a deeper understanding of analyzed cases and facilitate the development of concepts used in the research. Data were collected between March and December 2007.

### Data analysis

All interviews were recorded, transcribed verbatim and then analyzed according to recommendations by Strauss and Corbin [27,28], i.e., data collection and data analysis took place simultaneously in order to identify ideas, which then guided the next interview. The principal investigator and one of the Persian co-authors carefully read the texts to obtain an overall understanding of the full text. The interview transcriptions were then compared with the recorded digital files for accuracy. During the open coding phase, all the interviews were read several times, and key words or phrases, incidents and facts in the text were noted. During this phase, primary codes were extracted. The codes and data were compared for similarities and differences, and then categories and sub-categories were developed. All co-authors agreed upon the questions that would be raised and discussed their final wording after pre-test. Accordingly, from the first interview, a preliminary set of codes, categories and sub-categories was created and approved by the co-authors and research group. For the first four interviews, the principal investigator came back to the participants and checked the summary of understanding of the interview. Data collection process was continued until saturation of each concept was reached, and further data collection failed to contribute new information. During interviews, any identified concepts were discussed until saturation. After axial coding, and at the end of the selective coding phase, a core variable was identified. For that purpose all co-authors and research group members also agreed upon core variables that had been raised and discussed for their final decision.

### Ethical considerations

The study was approved by the National Ethics Committee of Iran [30]. Study participants were informed that their participation was confidential and voluntary and information used in the article would be anonymous. Information explaining the aim of the study was provided orally and in writing. The participants then signed informed consent or verbally consented to participating in the study, including being both interviewed and recorded.

## Results

"The lack of a system approach to road-user safety" was the core variable. Barriers in relation to RTI prevention were identified as: human factors (divided into two concepts of traffic safety culture and enforcement); transportation system (divided into two concepts of vehicle safety and infrastructure); and organizational coordination. Suggestions for improvement included education (for the general public and targeted group training), more effective legislation, more rigorous law enforcement, improved engineering in road infrastructure, and an integrated organization to supervise and coordinate preventive activities. (Table 1)

**Table 1:** Barriers in road traffic injury prevention

Categories	Human factor		Transport system		Coordination
**Sub categories**	**Traffic safety culture**	**Enforcement**	**Vehicle safety**	**Infrastructure**	**Organizational coordination**

**Codes**	Lack of road safety knowledge	Lack of strengthened traffic law legislation	Insufficient vehicle safety	Lack of separate lanes for low speed vehicles	Lack of an overall agency to coordinate road safety policy
	Lack of compliance with traffic law	Lack of sufficient authority for traffic police	Lack of vehicle safety standards test	Lack of safe routes for walking and cycling	Lack of coordination among organizations
	Lack of knowledge of traffic law	Lack of modern equipment for traffic police monitoring	Old car fleet	Insufficient road repairs	Parallel activities for some tasks
	A sense of urgency in road-users	Too easy to get driving license	Incompatibility of the vehicles on the road	Many accident black spots	Interaction in the duties of the other organizations
	Driving without caution	Old fashioned traffic monitoring by police	Difficulty in motorcycle visibility	Inadequately separated roads	Lack of sustainability of the preventive plan
	Insufficient driving training prior to licence	Low fines for traffic offences	Non-standard motorcycles	Non-standard roads	Lack of comprehensive injury surveillance
	Lack of confidence in motorcycle helmet use	Difficulty in confiscating vehicles from offenders	Lack of attention to child safety equipment	Lack of guard rails on dangerous roads	Excessive workload for traffic police dealing with drivers
	Lack of attention to seat belt use	Public's lack of confidence in the police	Lack of vehicle air bags	Inadequate traffic signs on the roads	Lack of a "National Decision" for RTI prevention
	Traditional wearing of dark clothes	Lack of sustainability of the preventive plan	Lack of ABS	Insufficient road lighting	Lack of enough research activities
	People's expectations of leniency from police when offending		Substandard motorcycles		Lack of reliable RTI data
	Low attention to traffic calming		Lack of standard equipment in vehicles		Inadequate public transport

### Human factors

#### Traffic safety culture

All participants pointed out that the current traffic culture of road-users and their safety behaviour are the most important barriers to RTI prevention. Undeveloped traffic culture was particularly pronounced among people who lived in the countryside. Traffic culture was regarded as "knowledge, values and beliefs about safety behaviour and compliance with traffic safety rules".

*(P3) Bad driving is a problem in our country. Most car drivers like to drive fast without attention to safety behaviour. (P7) While helmet wearing and seat belt use are compulsory in the country, most motorcyclists don't wear a helmet and it seems they don't believe it*....

Most participants pointed out that there is a lack of compliance with traffic law among road-users. Accordingly, some parameters identified in undeveloped traffic safety culture related to: a lack of knowledge of and confidence in traffic law; a sense of urgency in most road-users, which is reflected in their driving behaviour. Moreover, a high volume of trips by private cars were regarded as other barriers affecting road-user safety.

*(P2) Most people, especially those living in the countryside, don't know about traffic regulations and they don't have enough information about traffic rules... (P9) young motorcyclists usually don't wear a helmet and some of them don't have a driving licence*...

Concerning improving road-users' behaviour, most participants were of the opinion that education is the key to RTI prevention. As they pointed out, this should take the form of campaigns directed at the general public and training targeted at specific groups. The focus of this education should be on changing people's behaviour. A number of suggestions came up e.g.: encouraging calmness and patience among all road-users, increasing knowledge in road-user safety, and introducing traffic calming measures including more and lower speed limits. The mass media, especially television, should be used to achieve many of these goals. However, some professional in the health sectors pointed out that the importance of improvements to the infrastructure rather than simply a focus on road-user behaviour changes could be more effective. On the other hand, target group training was also recommended. This included a more comprehensive training course including road safety training prior to the driving test that needs to be followed up by continuous training, especially for professional drivers and those drivers who are illiterate.

*(P16) Traffic safety culture is a problem in the country in relation to road traffic accidents...the best way for its prevention is public education campaigns using mass media, especially television*...

#### Enforcement

According to participants, lack of enforcement was the other important obstacle to safe behaviour among motorized road-users. Moreover, some participants pointed out that traffic violations in Iran are usually punished by imposing fines. For dangerous behaviour, vehicles can be confiscated or driving licenses revoked; however in practice this is difficult. Accordingly, they mainly focus on imposing fines, which however, are relatively low. All participants felt this problem could be addressed by strengthening the legislation, law enforcement and providing modern equipment for traffic police monitoring.

*(P4) We believe that the current authority for law enforcement is not adequate and it is difficult for us to punish traffic offences. Since revoking a driving license is a very difficult process, we prefer to not do that*...

Most participants pointed to the lack of detailed instructions given by traffic police; old-fashioned police monitoring of traffic behaviour on interurban roads; and a lack of adequate public cooperation with the traffic police as other concerns. Moreover, as expressed by most participants, the legislation needs to be strengthened, the level of fines and other sanctions should be increased, and the issuing of driver's licenses should be more restricted with a greater focus on driving training and skills. However, participants in police office pointed out that during the recent new policy has led to the traffic police being more restrictive in the issuing of driving licenses.

*(P14) The current station-based police monitoring of traffic management is out of date and old fashioned; the laws should provide greater authority for police. The main form of punishment for traffic offences is fines which are too low... (P24) It is necessary to install more speed cameras for traffic monitoring*.

### Transportation system

#### Vehicle safety

As pointed out by a number of participants, two important obstacles related to vehicles were identified. Firstly, there is insufficient attention to car fleet safety; there is a lack of standards as regards the safety and crashworthiness of vehicles in the car industries. Secondly, there has been an over-production of vehicles in recent years with insufficient attention to crashworthiness and design safety. As expressed by all participants, a large number of old, even less safe vehicles that are incompatible in terms of speed with newer cars, was another important barrier.

*(P2) In recent years a new loan-to-buy system has been implemented, making it much easier to buy a car and resulting in a dramatic increase in both the passenger car fleet and motorized two wheeled vehicles*.

According to most participants, some other concerns related to vehicles were the shortcomings in public transport, and a lack of well-designed seat belts and child car seats. Most professional participants pointed out that a lack of air bags and anti-block braking systems (ABS) in most vehicles, very poor safety standards in motorcycles as well as a lack of safety equipment were the other obstacles to vehicle safety. These factors, hand in hand with very cheap petrol and a high volume of trips by private cars, could indirectly make the situation worse.

*(P12) In the car industry we think of everything, but little on safety. Petrol is cheaper than water! Which makes the situation even worse*...

Suggestions to deal with vehicle safety, expressed by professional participants, included vehicle safety improvements in the car industry and the implementation of research activity for vehicle safety indicators. The participants also suggested that the removal of old vehicles from the roads needs to be more facilitated and periodic vehicle inspection needs to be more rigorous.

*(P23) An old car fleet is one important problem. Not only they are unsafe themselves but they are also a danger to other vehicles on the road and we should provide more incentives to remove them from the vehicle fleet*...

#### Infrastructure

According to all participants, a large number of problems in relation to the transport system and its safety came up, mainly relating to the one way streets, inadequate road lighting, a lack of special roads for low-speed vehicles, a lack of enough roadside protection, lack of sufficient traffic signs on the roads, a poor overall level of road maintenance and repair as well as a large number of accident black spots. Moreover, a lack of safe routes for pedestrians and cyclists, and incompatibility of high- and low-speed vehicles were other obstacles. As expressed by participants, more traffic signs, quicker road repairs, installation of speed bumps in heavily populated areas and crash barriers in high-risk areas, and more alternative road junctions will all help to reduce crashes at black spots in both the short and the long term. Furthermore, most participants in road administration office and police officers as well as EMS pointed out that a better urban infrastructure including splitting roads up into different lanes, making them wider, and reserving special lanes for emergency services would also help matters.

*(P17) There are many accident black spots on the roads throughout the country ... (P21) Traffic police have made substantial efforts to improve road safety, however, due to poor vehicle and road safety design, the number of accident and casualties is still high*...

### Organizational coordination

Most participants raised the issue of inadequate communication and coordination between organizations in relation to road safety activities, which was one of the most important obstacles to RTI prevention. As participants pointed out, there are currently several organizations working in the road safety field but they are poorly coordinated. Accordingly, there is no single agency with overall authority and responsibility to make decisions and arrange road safety activities. Some other examples of the lack of coordination, expressed by participants were: the same task being carried out by two organizations; lack of a clear allocation of responsibility for some activities; lack of sustainable planning with regard to road safety activities among sectors. Moreover, participants in health sectors and road administration office as well as police office pointed out that there is no comprehensive information system to register motoring offences in Iran as whole, resulting in no distinction being made between traffic offenders and law-abiding citizens.

Most participants pointed out that the most important factor to decrease RTIs would be a "National Decision", whereby all sectors and road-users would decide to tackle this major national problem. As expressed by participants, it would be possible to devise a future plan and activities. Moreover, according to the participants, road-user safety could be improved by integrating safety activities and research.

*(P19) Currently different organizations have responsibility in road safety, but there is no defined organization to coordinate and follow these activities. This problem can result parallel activities for some duties and lack of responsibilities for some others*...

## Discussion

This study, the first of its kind in Iran, describes the major barriers to RTI prevention and the related recommendations of facilitators in the field based on stakeholders' perceptions. The participants in our study believed that the greatest gains would be achieved by changing road-users' behaviour by means of information, education campaigns and the more rigorous enforcement of regulations. Barriers related to the transportation system and the need for its development were also commented on but were given less emphasis.

In terms of RTI prevention, the areas for potential intervention in all society can be included: environmental modification of the infrastructure, greater attention to safety in vehicle manufacture as well as the promotion of safe behaviours through social marketing, legislation and law enforcement [31].

The perception of the current traffic safety culture as one major barrier was pronounced by most participants in this study. The forms of behaviour, as commented on by participants, could be due to: a lack of safety knowledge and traffic rules; a sense of urgency among most road-users; inadequate driver training and testing; insufficient enforcement of traffic and transport regulations, which together make the situation far worse. While safe road-user behaviour is one important component, however, changing such behaviour should not simply focus on education and enforcement. This idea is supported by a systematic review indicating that education programmes alone, in the case of child pedestrians safety, does not reduce the risk of motor vehicle collisions [32]. Concerning public education efficiency, studies in high-income countries have revealed that a decrease in crashes due to public education campaigns can occur only if they are clearly targeted on specific forms of behaviour, like seat belt use or helmet wearing [33]. In terms of enforcement, in a case control study in the capital city of Iran, a project imposing penalties on motorcyclists showed that more rigorous enforcement did not decrease the total incidence of traffic crash injuries, but did lead to a shift to less severe injuries during the enforcement implementation [34].

The manner of enforcement in Iran however is important. A lack of strengthened traffic legislation, a tendency for people to expect extremely lenient treatment from the traffic police, a low level of fines for traffic violations and a lack of long-term sustainable planning are important obstacles. These situations can lead a lack of effective enforcement. It is interesting to note that experience from high-income countries shows that if road-users had greater respect for road traffic legislation, the number of road crash fatalities could be almost halved [33]. According to Elvik et al. (2004), campaigns combined with increased police enforcement appear to be more effective than campaigns without [33]. For example in the case of helmet wearing in our study, uncomfortable helmets, as commented on by motorcycle participants, along with the substandard helmets currently in use are important obstacles that need to be considered. In such cases, more education would not be more effective. However, in such case, better helmet design and enforcement may be more effective for its use, but this needs to be measured quantitatively. It seems that focusing on public education and inadequate enforcement are important, but not sufficient.

Experience in Sweden reveals that changing road-user behaviour by means of environmental changes rather than education campaigns or police enforcement is more effective. In this area, an observational study revealed that seat belt reminders in the vehicle led to a significant difference in seat belt use in the cars with and without seat belt reminders [35,36]. The participants in our study didn't place much emphasis on environmental changes to improve human behaviour. Iranian traffic police have made use of both public education and enforcement in different areas in recent years. Car manufacturers also need to consider crashworthiness and the various safety technologies more seriously. Now there is a variety of very different kinds of vehicles on the roads in Iran and their incompatibility in terms of size, speed and safety constitutes is a major challenge. Speed is almost universally recognized as a major contributory factor to both the occurrence of RTIs and their severity [10]. In this respect, more rigorously enforced speed limits seem to be a higher priority than a focus on education. Furthermore, it is really important to increase public awareness about how high speed contributes to the severity of RTIs, a factor which seems not to be understood or is seen as important.

Participants in this study pointed out that an over-production of new cars combined with a lack of emphasis on safety in their design was an important obstacle. It is well known that as motorization increases, specially in LMICs, there will also be a rapid increase in the number of crashes and injuries [10]. Iran currently has the largest automobile industry in the Middle East and Central Asia [12], which could also affect road-user safety. The situation is exacerbated by the existence of many old cars in the fleet, combined with inadequacies in the public transport system, leading to a greater use of private vehicles, thus increasing the risk of crash and injuries. It is also important that the other components including road and vehicles and their interaction are not ignored. This idea is supported by findings in other settings [37], that the government and the car industry have a major responsibility in road safety, whereas it is much more common for the road-users to be held responsible. Moreover, research in biomechanics has shown that changes in the design of vehicles could greatly reduce the frequency and severity of injuries [38].

### System versus individual approach to RTI prevention

In general, there are two different approaches to RTI prevention: the individual and the system approach [39,40]. There is a tendency by researchers and practitioners to look for only one or a few elements [9]. Traditionally, many studies have focused on factors relating to driver errors, poor vehicles and the road environment instead of finding the reason for injury outcome, which could be found in many LMICs studies as well as Iran [14,41-48]. In our study many participants pointed out that the majority of the crash casualties were due to human error and the rest were due to road and vehicle factors. In contrast, a system approach would be mainly directed toward the crashworthiness of the road transport as a system [39]. The Haddon model [49] broadens the scope for intervention to reduce road crash injury in three event phases: pre-crash, crash and post-crash. The epidemiological triads of human, vehicles and environment and their interaction during each phase of a crash was also considered. Furthermore, the Swedish Road Administration Model has examined RTIs using a system approach [39]. This model considered the interaction between the three components of RTIs to classify fatal car crashes according to safety indicators and fatal outcomes. Of the three components in this model, the road was the one that was most often linked to a fatal outcome [39]. Clearly, safety design in the Swedish road transport system has been developed and the country has one of the best road safety records worldwide. This implies that to improve road-user safety in Iran, a change in the attitudes toward road-user safety of the Iranian stakeholders is needed. Moreover, to assist this aim, and as pointed out by some participants in the health sector, establishment of comprehensive injury surveillance is needed.

As expressed by most participants in this study, lack of organizational coordination was another important obstacle. According to WHO, prevention of RTIs is a shared responsibility and needs multisectoral collaboration [1,9]. Collaboration might take the form of research, information sharing, policy development, advocacy and capacity development. Lack of coordination was mentioned in this study as one important barrier, which was in line with a study in the case of post-crash events [23]. The reason for this could be the lack of an overall agency to coordinate road safety policy. An additional reason could be the lack of a sustainable plan for RTI prevention and parallel activities being carried out for some tasks. A specific agency in the government should be identified to guide and arrange national agency efforts, which is in line with the recommendation of Mohan et al. [9]. A movement for safety in the community is another important strategy, which currently is underway in three communities of Iran, including Arsanjan, Kashmar and Badakhshan [50]. The effectiveness of such a movement has been shown in many RTI prevention strategies [9] and seems it is a need to be expanded throughout the country.

### Some policy implications

Policy intervention including both short- and long-term strategies should be considered by the authorities. In the long-term strategies, traffic system development needs to be considered as regards both car manufacturers and the road system. Road safety development can include taking action regarding accident black spots as well as the improvement of vehicle safety standards with a focus on crashworthiness and research. Short-term interventions include: increasing penalties and tougher enforcement for dangerous driving behaviours, speed management (with high priority) and the more installation of traffic-monitoring cameras. Safety education programmes should focus on defining the behaviour and changing the attitude of all road-users.

### Limitation and strength of the study

This interview-based study gathered the opinions of various actors, including road-users and victims of RTIs, relating to the barriers to and possible facilitators of RTI prevention. As such, it is one of the few studies adopting a qualitative approach to highlight ways of improving the current situation. The results point to a number of crucial areas in need of improvement, and for which some strategies have been proposed.

As is the case in qualitative studies, the number of participants was relatively small, but all participants were experienced and knowledgeable and saturation was reached for all concepts. The data were even validated using constant comparison analysis, which means returning to the data in order to verify and develop the categories further. This method requires the researcher to take one piece of data and compare it to all other data in terms of similarity and difference [51]. In the same vein, the input from victims and road users can be regarded as an important contribution - and as an originality of the study.

## Conclusion

Iran ranks as one of countries with the highest rate of fatal RTIs in the world and there is currently a gap in the knowledge of and attitude towards RTIs among participants that needs to be addressed through educational campaigns and more research in the field of road-user safety. The attitude among stakeholders in relation to road safety activities needs to change. As other RTI prevention models have shown, instead of an individual component approach, there is a critical need for a system approach to RTI prevention considering the interaction between road-users, the road infrastructure and vehicles as well as integrated organizational coordination in Iran. Although the current educational campaign is an important step in behaviour changes, most focus should be placed on environmental changes. The focus of all activities should take place on road users' safety.

## Competing interests

The authors declare that they have no competing interests.

## Authors' contributions

DKZ has made substantial contributions to the conception and design of the study, and taken responsibility for and coordinated the acquisition of data, which he gathered and analyzed. He took an active part in the analysis of the data, in its abstraction and in the writing-up of the manuscript. HRK and LL contributed to the conception and design of the study. HRK was involved in the data collection process and took part in the data analysis. LL also took part in the writing-up and finalization of the manuscript. RM, AB and BH contributed to the study design, data acquisition, results interpretation and writing-up of the manuscript. All authors read and approved the final manuscript.

## Pre-publication history

The pre-publication history for this paper can be accessed here:

http://www.biomedcentral.com/1471-2458/9/486/prepub
